# From two putative inquilines to one definitive parasitoid: clarifying feeding ecology and species limits in Nearctic *Euceroptres* Ashmead, 1896 wasps (Hymenoptera: Figitidae)

**DOI:** 10.1093/aesa/saag028

**Published:** 2026-07-14

**Authors:** Guerin E Brown, Louis F Nastasi, Charles Davis, Nicolas Sierra, Corey J Lewis, Makella J Steffensen, Dane Sweezer, Andrew A Forbes

**Affiliations:** Biology Department, University of Iowa, Iowa City, IA, USA; Biology Department, University of Iowa, Iowa City, IA, USA; Department of Entomology, Pennsylvania State University, State College, PA, USA; Biology Department, University of Iowa, Iowa City, IA, USA; Biology Department, University of Iowa, Iowa City, IA, USA; Biology Department, University of Iowa, Iowa City, IA, USA; Biology Department, University of Iowa, Iowa City, IA, USA; Biology Department, University of Iowa, Iowa City, IA, USA

**Keywords:** Cynipidae, Cynipini, micro-CT, taxonomy, oak gall

## Abstract

Oak galls are structures of modified plant tissue, induced by an ovipositing female oak gall wasp (Hymenoptera: Cynipidae: Cynipini), that provide food and shelter for her progeny. Myriad other wasp species exploit galls, feeding on either the gall wasp larva (parasitoidism), the gall tissue (inquilinism/kleptoparasitism), or other parasites within the gall (hyperparasitism). Though many oak gall-associated wasps are now described, their feeding ecology often remains poorly documented, particularly in the species-rich communities across North America. Wasps in the genus *Euceroptres* (Hymenoptera: Figitidae) exemplify this problem, long having been assumed to be inquilines based on limited evidence. We used dissections, microcomputed tomography scanning, and DNA barcoding to discover that *Euceroptres* do not modify the internal composition of their host gall. We also found a *Euceroptres* larva feeding on gall-inducer larva within an unaltered gall wasp larval chamber. Thus, *Euceroptres* are parasitoids, not inquilines. While sequencing larval and adult *Euceroptres*, we also found that the 2 previously described species in eastern North America, *Euceroptres whartoni* Buffington and Liljeblad, 2008 and *Euceroptres primus* Ashmead, 1896, did not separate into distinct clades, suggesting they are a single species. Subsequent morphological comparisons support this finding, allowing the establishment of *E. whartoni* as a **syn. nov.** of *E. primus.*

## Introduction

Parasitic wasps are some of the most species-rich of all animals, but their species limits and ecological interactions are also among the least well-understood (Noyes 2012, Huber 2017, Forbes et al. 2018). The diverse parasite communities of oak gall wasps (Hymenoptera: Cynipidae: Cynipini) have proved rich systems for discovering new species boundaries and ecologies (Askew et al. 2013, Sheikh et al. 2022, Ward et al. 2022, 2024). Oak gall wasps lay eggs into the meristematic tissue of oak trees and induce the formation of galls—atypical structures of plant tissue that provide food and protection to the developing wasp larvae (Egan et al. 2018). Species-rich parasitic wasp assemblages frequently attack both the gall wasp larvae and the gall tissue itself, with up to 25 individual parasite species associated with single gall wasp species in the Nearctic (Joseph et al. 2011, Forbes et al. 2016).

Surveys integrating genetic tools have revealed that several oak gall-associated parasitic wasp genera are far more species-rich than was previously understood, especially in North American oak galls. The genus *Ormyrus* Westwood, 1832 is an exemplar: through rearing and DNA barcoding, the single morphological species *Ormyrus labotus* Walker, 1843 (Hymenoptera: Ormyridae) was found to be 16 to 18 species (Sheikh et al. 2022). Similar discoveries have been made for the oak gall-associated genera *Ceroptres* Hartig, 1840 (Hymenoptera: Cynipidae) (Nastasi et al. 2024, Ward et al. 2024), *Synergus* Hartig, 1840 (Hymenoptera: Cynipidae) (Ward et al. 2020), and *Sycophila* Walker, 1871 (Hymenoptera: Eurytomidae) (Zhang et al. 2022): all are rife with cryptic and undescribed species.

The feeding ecologies of many oak gall parasites are also understudied. Parasitism in oak galls generally takes 1 of 3 forms: consumption of the gall-inducer (parasitoidism), consumption of the plant tissue, often involving the formation of pseudo-galls inside the original gall (inquilinism/kleptoparasitism), or parasitism of other parasitoids or inquilines (hyperparasitism). As new species are discovered and named in this system, their feeding ecologies often remain unknown or are assumed to match those of close relatives. One barrier to a better understanding of parasite ecology is that most specimens are reared from galls and are thus only observed as adults, after their interaction within the gall is complete.

*Euceroptres*  Ashmead, 1896 (Hymenoptera: Figitidae) is a genus known from North American oak galls but is an apparent rarity among oak gall parasitic genera in its low species richness. There are just 4 described species: 2 in the western United States, *Euceroptres montanus*  Weld, 1926 and *Euceroptres maritimus*  Weld, 1926, and 2 eastern species, *Euceroptres primus*  Ashmead, 1896 and *Euceroptres whartoni*  Buffington and Liljeblad, 2008. The 2 western species are known associates of only 1 oak gall wasp species each: *E. montanus* from *Disholandricus truckeensis* (Ashmead, 1896) and *E. maritimus* from *Callirhytis quercussuttoni* (Bassett, 1881). Both host galls take the form of woody stem swellings. The 2 eastern species are only separable by a single morphological diagnostic character: *E. primus* has a short, notch-like median mesoscutal impression, while *E. whartoni* has an elongate median mesoscutal impression, which extends across at least one-fourth of the mesoscutum (Buffington and Liljeblad 2008). Rearing records suggest that eastern *Euceroptres* species have at least 6 oak gall hosts (Ward et al. 2022), primarily leaf swellings found on trees in sections *Quercus* (white oaks) and *Lobatae* (red oaks). Whether there are more species of *Euceroptres* that remain to be discovered, or what their respective host gall ranges are, are open questions. We hypothesized that eastern North American *Euceroptres* are substantially more species-rich than existing taxonomic descriptions suggest, especially given the use of numerous hosts across multiple *Quercus* sections.

The feeding ecology of *Euceroptres* is also in question; the literature is highly variable in discussing the role of these wasps inside their host galls (Table 1). Upon describing the genus and the first known species, Ashmead (1896) offered no commentary on the possible life history of *Euceroptres*. Soon after, Weld (1926) provided the first suggestion that they may be inquilines, referring to the newly described species *E. maritimus* and *E. montanus* as “guest flies”; this term was commonly used to refer to inquilines in cynipoid literature in the late 19th and early 20th centuries. Inquilines are phytophagous, invading the gall and feeding on internal plant tissue subsequent to initiation of gall formation by the gall inducer (Sanver and Hawkins 2000). Ashmead and Weld both considered *Euceroptres* to be a member of Cynipidae, which includes only phytophagous species, thus supporting the early assumption of an inquiline life history. Ronquist (1994, 1999) further supported the status of *Euceroptres* as inquilines, referring to them as “figitoid inquilines” along with various other gall-associated Figitidae. Unlike prior studies, Ronquist (1994, 1999) employed phylogenetic inference in this assessment, finding species most closely related to *Euceroptres* to be phytophagous and suggesting, through parsimony, that *Euceroptres* was an inquiline. However, Ronquist and Nieves-Aldrey (2001) moved *Euceroptres* from Cynipidae to Figitidae, a group that is currently known to be dominated by parasitoid life histories. More recent studies began to refer to *Euceroptres* as parasitoids (eg Buffington 2008, Buffington and Liljeblad 2008, Paretas-Martínez et al. 2011; Table 1). However, there is no detailed study of the feeding ecology, and the shift toward a parasitoid hypothesis was largely unaccompanied by conclusive evidence (Ronquist et al. 2018). More recently, Blaimer et al. (2020) performed ancestral state reconstructions of feeding ecologies, which support dominance of parasitoidism in Figitidae, further supporting a parasitoid hypothesis for *Euceroptres*. The confluence of these prior studies led us to hypothesize that *Euceroptres* are truly entomophagous parasitoids rather than phytophagous inquilines, and we sought to empirically test this hypothesis.

**Table 1.** Discussion of *Euceroptres* life history in Cynipoidea literature

Author	Year	Life history description	Comments
**Ashmead**	1896	No comment on biology	Described genus *Euceroptres* and type species *E. primus*
**Weld**	1926	Inquiline (“guest”; see text)	Described *Euceroptres maritimus* and *E. montanus*
**Weld**	1952	Inquiline	
**Ritchie**	1984	Inquiline	
**Ronquist**	1994	“Figitoid inquiline”	
**Ronquist**	1999	“Figitoid inquiline”	
**Ronquist and Nieves-Aldrey**	2001	“Figitoid inquiline”	
**Buffington**	2008	“Formerly referred to as figitoid inquilines”	
**Buffington and Liljeblad**	2008	Parasitoid	Described Euceroptresinae; revised *Euceroptres*, describing *E. whartoni*
**Paretas-Martínez** et al.	2011	Parasitoid	
**Buffington** et al.	2012	Parasitoid	
**Ronquist** et al.	2015	“Presumably… parasitoids”	
**Ronquist** et al.	2018	“…no conclusive evidence that [*Euceroptres*] are parasitoids”	
**Blaimer** et al.	2020	Parasitoid/unknown	Scored *Euceroptres* as both “parasitoids” and “unknown” in ancestral state reconstructions of cynipoid life histories
**Buffington** et al.	2020	Parasitoid or inquiline	
**Ward** et al.	2022	“…not known in detail”	

To address both the feeding ecology and species-richness of *Euceroptres* parasites, we collected oak galls from multiple locations on various oak tree species. Insects were reared, and a subset of galls were dissected or scanned using a microcomputed tomography (micro-CT). Emerging adult or dissected larval *Euceroptres* specimens were then sequenced for mitochondrial COI DNA barcodes (mtCOI), and a subset of these were selected for ultraconserved element (UCE) sequencing.

## Methods

### Collections and Rearing

Collections were made in 2 distinct stages: opportunistic collections across North America, followed by species-specific targeted collections. Opportunistic collections took place in 32 continental states in the United States and parts of Canada and Mexico from 2015 to 2025, encompassing 570 gall morphospecies across approximately 4,000 individual collection events. This work is part of a larger effort to document the interactions, host associations, and biodiversity of North American oak gall communities. Collections were conducted by both our research team and a network of citizen naturalists from across North America. We identified galls and their host tree to morphospecies, photographed them, and stored galls in labeled plastic bags alongside other galls of the same morphospecies from the same individual tree. Galls were then placed in small plastic cups and maintained in a climate-controlled incubator with standard temperature, relative humidity, and light cycle conditions (Supplementary Table S1). We checked containers daily and preserved all emerging insects in 95% EtOH for identification and DNA extraction. Ward et al. (2022) summarize collections from 2015 to 2019, wherein a total of 803 *Euceroptres* from 6 gall species were recorded. A subset of these specimens was used to inform this study.

More than 90% of all *Euceroptres* from previous collections were recorded emerging from galls induced by *Andricus quercuspetiolicola* (Bassett, 1863). For our feeding ecology studies, we conducted targeted collections of *A. quercuspetiolicola* galls each spring from 2023 to 2025. We reared galls of *A. quercuspetiolicola* collected in 2023 for adult wasps, while for later collections, we prioritized larval dissections and micro-CT scans.

### Microcomputed Tomography

To study the internal structure of *A. quercuspetiolicola*, both in the presence and absence of *Euceroptres*, we scanned galls using micro-CT. Three galls were chosen from among 2,023 collections after most insects had emerged. These included 1 gall from which only *A. quercuspetiolicola* had emerged, which served as our control, and 2 galls from which *Euceroptres* had emerged. This design allowed us to assess whether galls with *Euceroptres* had chambers that differed in size, shape, or orientation from those in “normal” galls, which would indicate an inquiline habit.

To prepare galls for scanning, we flash-froze them in liquid nitrogen and then stored them at −20 °C. Galls were transported in a cooler to the University of Iowa’s Materials Analysis, Testing, and Fabrication (MATFab) facility and scanned using a SkyScan 1272 micro-CT scanner (Bruker microCT, Kontich, Belgium). Scanning settings varied slightly between samples depending on the size of the gall, but all scans were between 13 and 25 mm from the X-ray source with no filter. Shadow images were reconstructed into 3-dimensional CT data using NRecon (Bruker microCT). During reconstruction, we corrected each scan for post alignment, beam hardness, and ring artifacts. Following reconstruction of micro-CT images, we stylized the data in 3D-Slicer (Fedorov et al. 2012).

After loading data into 3D-slicer, the gall was isolated from most of the surrounding airspace using an “endocast,” which includes both the gall, the immediate surrounding airspace, and the internal space. Next, the physical tissue was isolated from the airspace using thresholds of material density. Airspace was separated manually into both external and internal space by manual pruning. We then compared the location, orientation relative to the center of the gall, and approximate position of cells from each gall to determine if there was evidence of inquiline presence.

### Dissections

To study larval interactions inside the gall and determine where *Euceroptres* larvae might be found, we dissected 45 *A. quercuspetiolicola* galls. Dissections were performed under a Leica S9i stereo microscope (Leica Microsystems, Heerbrugg, Switzerland). Dissection equipment was sterilized in 10% bleach for at least 10 min before being used. Galls were then positioned on a makeshift stand of plasticine clay, and individual slices of the gall were taken every 2 mm starting from the apex of the gall down to its base. An image of the gall was collected following each slice. Any larvae found inside the gall were carefully removed with sterilized forceps and preserved in 95% EtOH. Larvae found interacting inside a single gall chamber were recorded. In total, 162 larvae found in gall chambers during dissections were extracted and sequenced.

### Genetic Identification

Larvae and adults were barcoded for genetic identification. DNA extraction was performed using a cetyltrimethylammonium bromide and phenol/chloroform method (Chen et al. 2010). We performed PCR for mtCOI using degenerate universal primers, Deg-COIF (5'-RKTCAACAAATCATAAAGATATTGG-3') and Deg-COIR (5′-TAAACTTCWGGGTGWCCAAAAAATCA-3′). To allow for multiplexing, primers had 13 bp barcoded tags, adapted from Srivathsan et al. (2021), added to the 5’ end of the molecule. PCR conditions were: 95 °C (2 min), 35 cycles of (95 °C [15 s], 45 °C [15 s], 72 °C [30 s]), 72 °C (1 min).

mtCOI amplicons were pooled and sequenced on an Oxford Nanopore MinION Mk1B platform with a v14 (SQK-LSK114) Ligation Sequencing Kit (Wang et al. 2021). We sequenced using a R10.3 chemistry Flongle Flow Cell. Sequencing reads were demultiplexed using a custom SnakeMake workflow (Mölder et al. 2021). The pipeline first demultiplexes reads using minibar v0.24 (Calacademy-Research/Minibar: Dual Barcode and Primer Demultiplexing for MinION Sequenced Reads), followed by dereplication, clustering, and chimera removal using VSEARCH v2.30.0 (Rognes et al. 2016), which uses the UCHIME2 algorithm for denoising and chimera removal. The computational process, and all necessary scripts, are documented by Brown et al. (2025). At this stage, some highly divergent and clearly pseudogenized nuclear mitochondrial DNA sequences were discovered and removed. We supplemented our dataset with an mtCOI sequence extracted from *E. montanus* UCEs from Blaimer et al. (2020) to capture all 4 named Nearctic *Euceroptres*. We aligned sequences from denoised clusters using the MUSCLE algorithm (Edgar 2004) in Uniprot’s Ugene v50 (Okonechnikov et al. 2012) before computing a gene tree using IQ-Tree v2.0.7 (Minh et al. 2020) using ModelFinder (Kalyaanamoorthy et al. 2017) with 5,000 ultra-fast bootstraps and an approximate likelihood ratio test (aLRT; Guindon et al. 2010). The unpruned gene tree (Supplementary Fig. S1) was stylized using the “ape” package in R 4.3.3 (Paradis and Schliep 2019, R Core Team 2024).

We sequenced UCEs for a subset of 12 specimens representing the morphological and host diversity from our collections. Nine were prepared and sequenced at the University of Iowa. The remaining 3 were sequenced as part of a larger study on Cynipoidea *sensu lato* by a collaborating laboratory at The Pennsylvania State University (Nastasi 2025) using Rapid Genomics LLC (Gainesville, Florida, United States). For samples that were prepared in-house, we began with a 15-min enzymatic fragmentation to produce 300 to 600 bp fragments. Next, we performed end repair, end adenylation, adapter ligation, and PCR enrichment for libraries using a KAPA HyperPlus kit. Pools of equimolar samples were enriched with UCE probes (Faircloth et al. 2015, Branstetter et al. 2017) using a myBaits UCE Hymenoptera 2.5Kv2p kit (Arbor Biosciences). Finally, these were sequenced on an AVITI24 platform (Element Biosciences) with a paired-end 2×150 bp format. We processed sequence data using Phyluce v1.7.3 (Faircloth 2016) to cluster and align individual UCEs. Finally, we inferred the phylogeny with a 90% matrix of 1,445 loci using IQ-Tree v2.0.7 with ModelFinder. Nodal support of the final UCE phylogeny was calculated using 1,000 ultra-fast bootstraps, an aLRT, and an ultra-fast bootstrap optimization by nearest neighbor interchange to reduce overestimation of branch supports (bnni; Hoang et al. 2018). The unpruned UCE tree (Supplementary Fig. S2) was stylized using the same process as described for the mtCOI gene tree.

### Morphological Examination

To examine morphological variation between *Euceroptres* species, several characters were measured or scored for 79 adult female specimens reared from a variety of host galls (Supplementary Table S2). Examined specimens were air-dried and point mounted. Specimens were first confirmed as *Euceroptres* using Buffington et al. (2020), then evaluated based on morphological descriptions given by Buffington and Liljeblad (2008). We deposited 35 examined and 3 additional *Euceroptres*, including 1 *E. maritimus*, at the University of Iowa Museum of Natural History (SUI: INS: 49790-49827); the remaining 39 are deposited at the Frost Entomological Museum (PSUC; University Park, Pennsylvania, United States). Digitized label data of all examined material, following Darwin Core biodiversity data standards (Weiczorek et al. 2012), are available in Supplementary Table S4. For each *Euceroptres*, the total body length in lateral view was recorded in millimeters along with the ratio of the length of the mesoscutal medial impression to the length of the mesoscutum as measured in dorsal view. The remaining 2 specimens were the holotypes of *E. primus* and *E. whartoni*, for which measurements were taken from photos available on the US National Museum of Natural History’s type specimen database (specimens USNMENT00802259 for *E. primus* and USNMENT00802423 for *E. whartoni* accessible at https://collections.nmnh.si.edu/search/ento/). Lastly, the general body coloration of each specimen was scored as light brown, dark brown, or black; substantial but consistent color variation was obvious during our initial observations of *Euceroptres* wasps despite this not being reported in the last revision of the genus (Buffington and Liljeblad 2008). Analysis on the data was performed in R 4.3.3.

## Results

### Rearing and Host Associations

Previous work (Ward et al. 2022) resulted in 803 *Euceroptres* parasites from 5,533 galls, representing 6 gall morphospecies. Collections from 2023 to 2025 reared a total of 253 *Euceroptres* parasites from 1,351 galls across 9 oak gall species. In total, our collection efforts reared 1,056 *Euceroptres* from 6,884 galls across 9 host gall species (Table 2). These included an association with *Neuroterus saltarius*  Weld, 1926 not previously reported in the literature.

**Table 2.** Rearing records for *Euceroptres* collected from 2015 to present showing host gall species, the number of *Euceroptres* reared from each host, and the total number of galls collected per host morphospecies

Host gall species	Gall generation	# ***Euceroptres***	# Galls collected	Geography	Gall morphology	*Quercus* sect.
***Andricus foliaformis* Gillette**	Sexual	34	280	Eastern	Integral, polythalamous, succulent leaf lamina swelling	*Quercus*
***Andricus quercuspetiolicola* (Bassett)**	Sexual	931	1,611	Eastern	Integral, polythalamous, succulent leaf petiole swelling	*Quercus*
***Bassettia flavipes* (Gillette)**	Sexual	9	464	Eastern	Integral, polythalamous, succulent leaf midrib swelling	*Quercus*
***Callirhytis quercusfutilis* (Osten Sacken)**	Sexual	5	487	Eastern	Integral, polythalamous, succulent leaf lamina swelling	*Quercus*
***Callirhytis quercussuttoni* (Bassett)**	Sexual	7	1	Western	Integral, polythalamous, woody stem swelling	*Lobatae*
***Callirhytis scitula* (Bassett)**	Sexual	20	111	Eastern	Integral, polythalamous, woody bud swelling	*Lobatae*
***Druon quercusflocci* (Walsh)**	Asexual	1	79	Eastern	External, polythalamous, hairy leaf vein growth	*Quercus*
***Melikaiella ostensackeni* (Pujade-Villar et al)**.	Sexual	33	1,107	Eastern	Integral, polythalamous, succulent leaf lamina swelling	*Lobatae*
***Melikaiella tumifica* (Osten Sacken)**	Sexual	11	94	Eastern	Integral, polythalamous, succulent leaf petiole swelling	*Lobatae*
***Neuroterus saltarius* Weld**	Asexual	5	2,650	Eastern	External, monothalamous, spangle leaf galls	*Quercus*

The 7 *Euceroptres* reared from the west coast gall *C. quercussuttoni* (Bassett) were all *E. maritimus*, while *Euceroptres* reared from other galls (all in the eastern or midwestern United States) were morphologically either *E. whartoni* or *E. primus*. Note that *Disholandricus truckeensis*, the host of *E. montanus*, is not included as we did not collect this species.

### Feeding Ecology

**Fig. 1.**
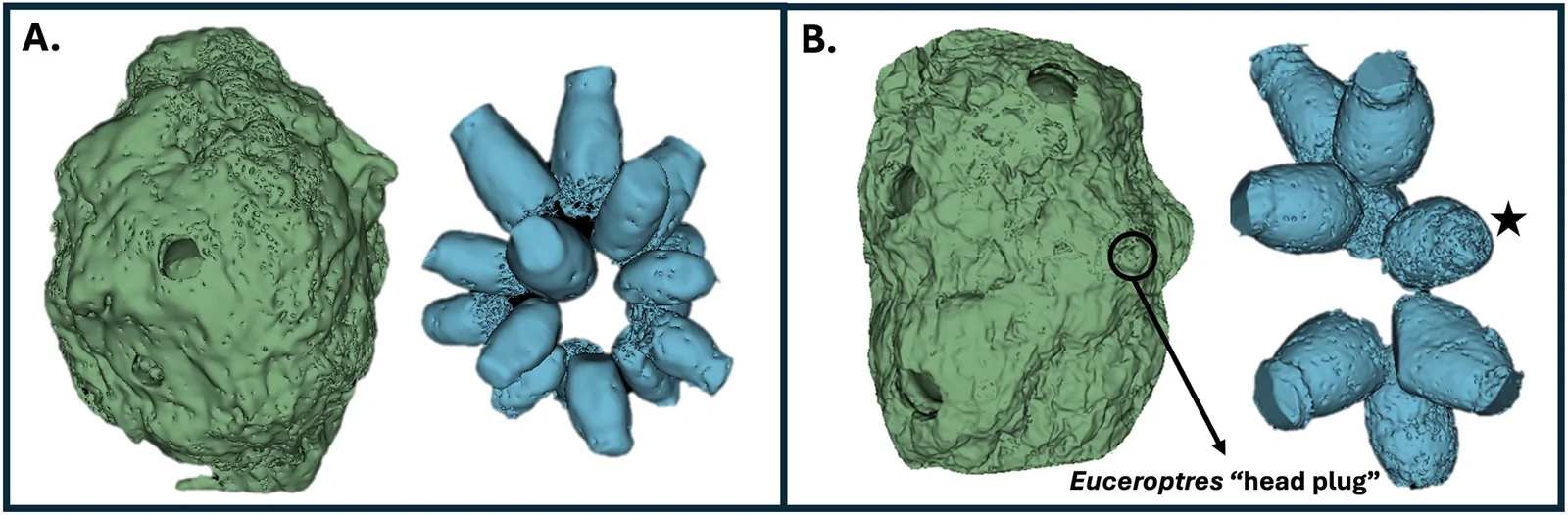
Micro-CT scans of *A. quercuspetiolicola* galls. (A) Gall from which only the gall inducer emerged, showing larval chambers (blue) radiating out from the center. (B) Gall from which primarily *Euceroptres* emerged, also showing larval chambers radiating from the gall’s center; the circle indicates the head of a *Euceroptres* that had died before fully emerging from the gall, and the star marks the corresponding larval chamber. For each panel, the external gall shape is shown in green (left) and isolated internal larval chambers in blue (right).

**Fig. 2.**
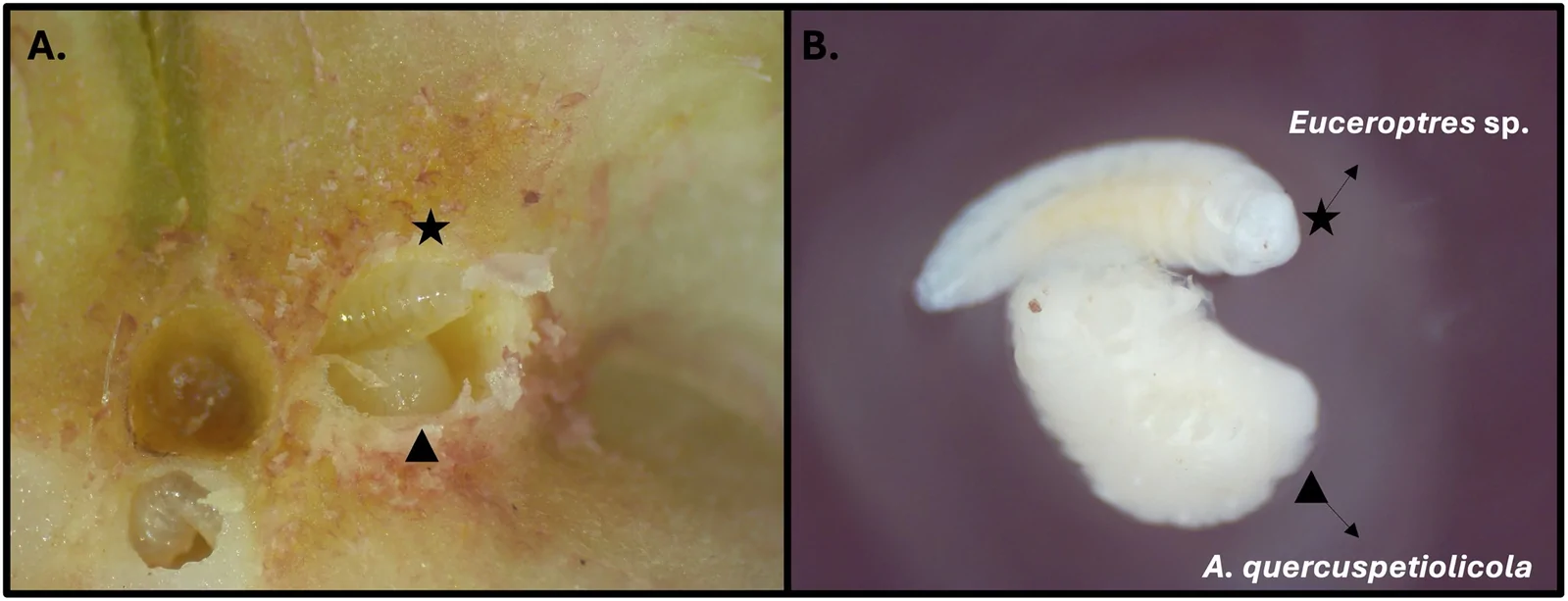
Images from a dissected *A. quercuspetiolicola* gall. (A) Two larvae within a single larval chamber; the upper larva (star) was observed actively feeding on the lower larva (triangle). (B) The same larvae after removal. MtCOI sequences confirmed the upper larva as *Euceroptres* and the lower larva as the gall inducer, *A. quercuspetiolicola*.

Comparisons of the micro-CT scans from galls with and without *Euceroptres* showed no signs of additional cells that differed drastically in shape from those produced and inhabited by the gall inducer (Fig. 1). A dead *Euceroptres* adult was also discovered inside what looked like a normal gall inducer’s chamber. Serial dissections of *A. quercuspetiolicola* produced 162 larvae. Most dissected larvae proved to be gall inducers or other parasites, but 4 were *Euceroptres*, and all were found in a gall inducer’s chamber. One *Euceroptres* larva was found coiled around and feeding on a second larva, which DNA barcoding revealed to be *A. quercuspetiolicola* (Fig. 2).

### Genetic Identification and Diversity

**Fig. 3.**
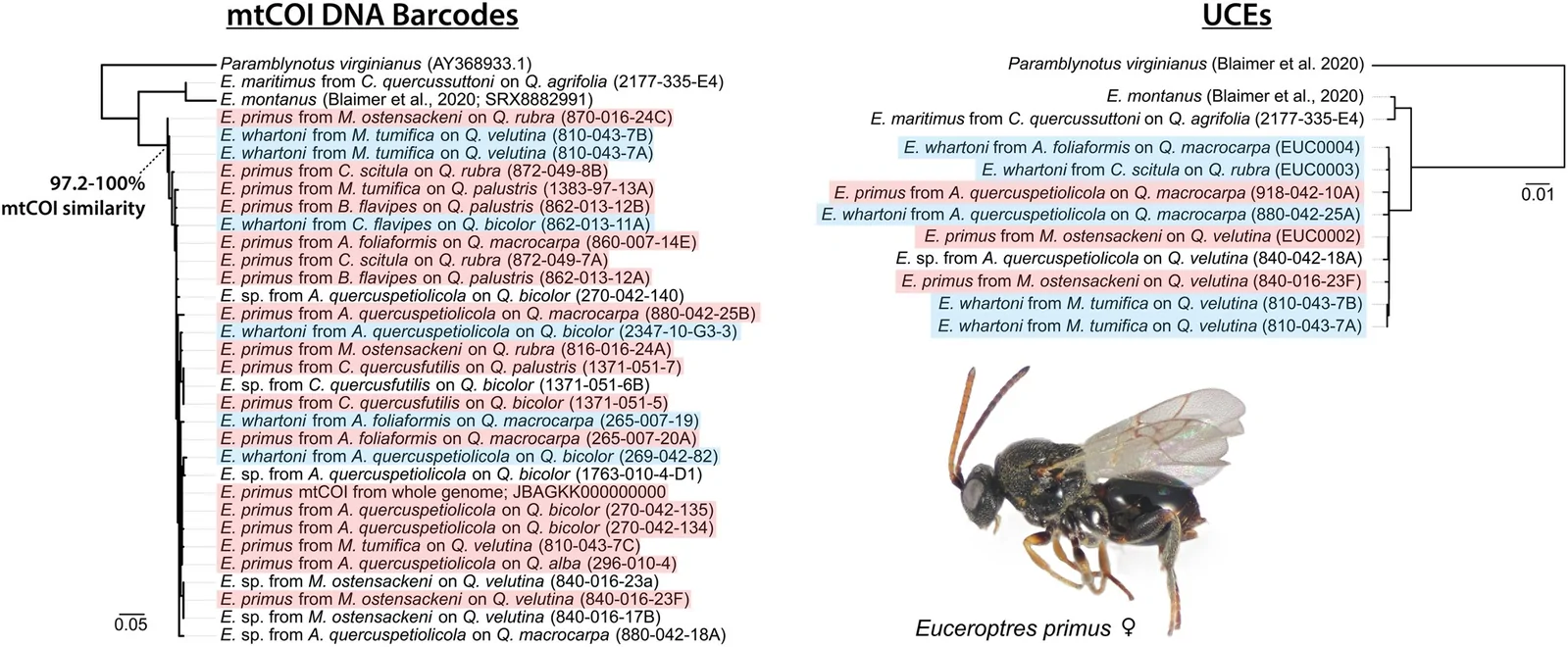
Pruned and stylized mtCOI maximum likelihood phylogeny (left) and stylized UCE phylogeny (top right) of *Euceroptres*. Tip labels for both trees are highlighted by morphological identification: red indicates specimens consistent with *E. whartoni*, blue indicates specimens consistent with *E. primus*, and uncolored labels within the large monophyletic clade containing both species did not fit the description of either. For both phylogenies, sequences for *E. montanus* and the outgroup, *Paramblynotus virginianus* Liu, Ronquist, and Nordlander, 2007, were acquired from previously published data.

The mtCOI and UCE trees both support a single-species hypothesis for *E. whartoni* and *E. primus*. The mtCOI gene tree groups *E. whartoni* and *E. primus* into 1 clade, which is sister to *E. montanus* and *E. maritimus* (Fig. 3). This clade is not subdivided by morphospecies, and sequences are 97.2% to 100% similar. *Euceroptres montanus* and *E. maritimus* sequences were 95.4% similar to each other and 87.7% to 91.5% similar to the *E. whartoni/E. primus* clade. This pattern is corroborated by the UCE tree, which also recovers a single clade of intermixed *E. whartoni* and *E. primus* that is sister to *E. montanus* and *E. maritimus* (Fig. 3).

### Morphology and Taxonomy

**Fig. 4.**
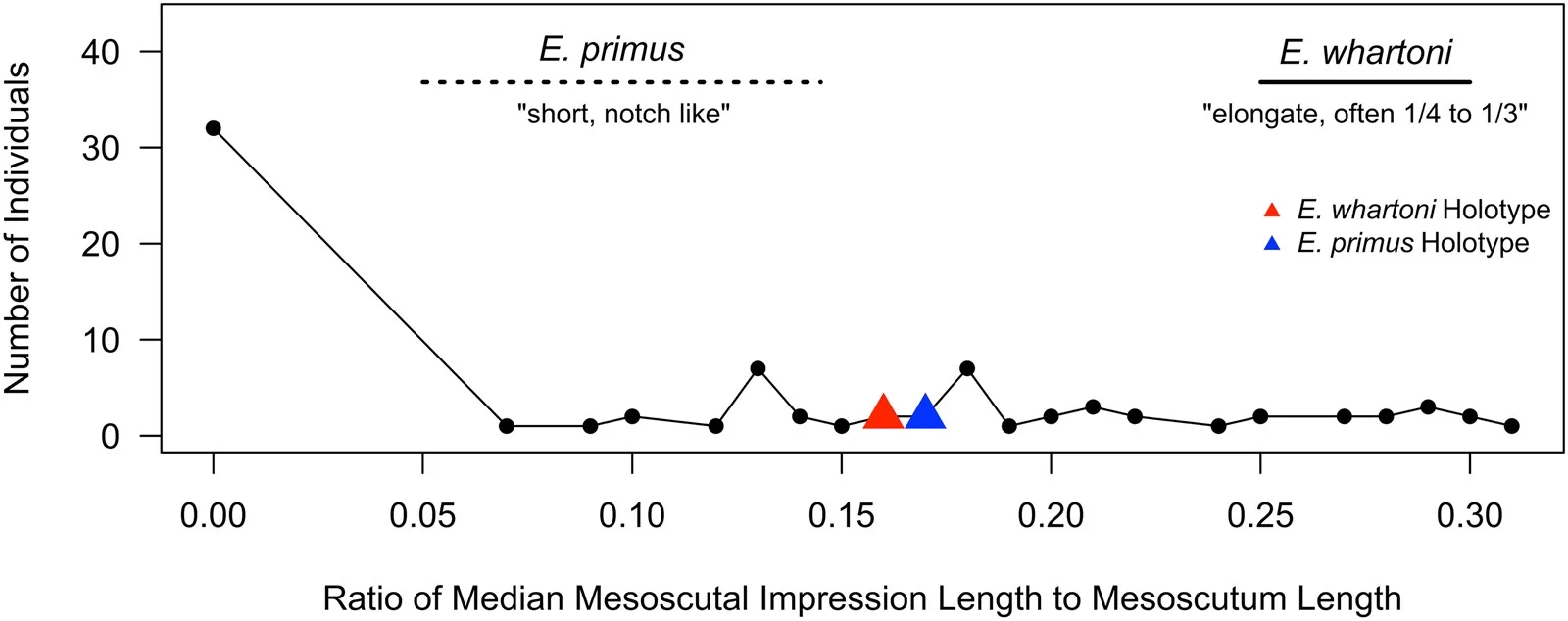
Distribution of the ratio of median mesoscutal impression length to mesoscutum length across 79 *Euceroptres* specimens. Ranges consistent with the descriptions of *E. primus* and *E. whartoni* are indicated above the graph. Triangles mark holotype measurements: red for *E. whartoni*, blue for *E. primus*.

**Fig. 5.**
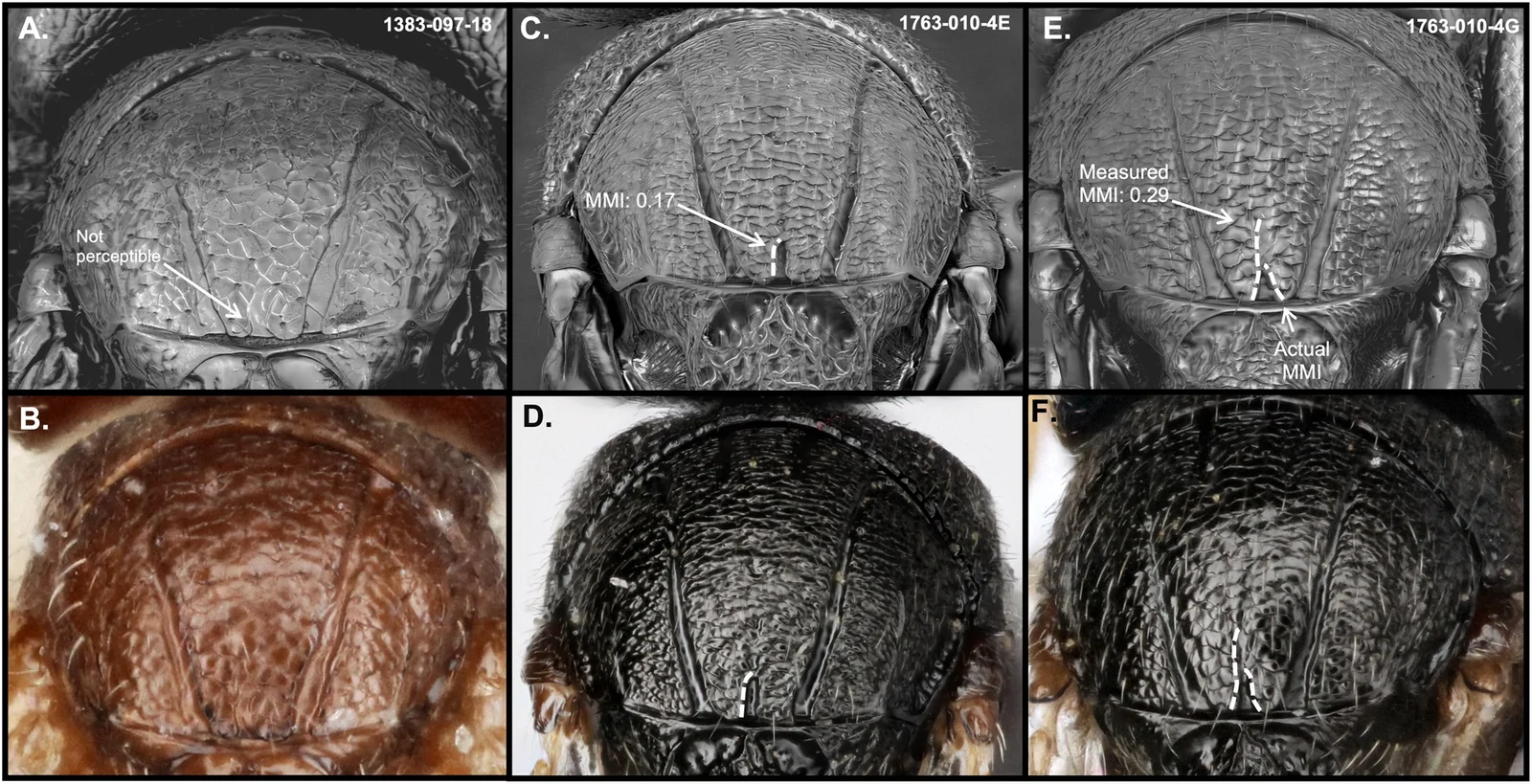
SEM images (top) and stacked photographs (bottom) of the dorsal thoraces of 3 *Euceroptres* specimens, illustrating the range and measurement challenges of the median mesoscutal impression (MMI). (A and B) Specimen 1383-097-18, which has no observable MMI. (C and D) Specimen 1763-010-4E, which has a short but observable MMI. (E and F) Specimen 1763-010-4G, which has a MMI that varies by imaging method. SEM images (E) show a short MMI, whereas light reflection (F) from anterior sculpturing in the photograph is traceable further along the mesoscutum and creates the illusion that the MMI extends further.

Among 79 measured individuals in the *E. primus*/*E. whartoni* clade, the ratio of the median mesoscutal impression to the length of the mesoscutum ranged from 0.0 (not perceptible) to 0.31. Furthermore, the holotypes of *E. primus* and *E. whartoni* are barely distinct in their presentation of the median mesoscutal impression, differing in the ratio of the impression to the mesoscutum length by only 0.01. This suggests that the holotypes of *E. primus* and *E. whartoni* are conspecific based on the criteria for species diagnosis presented by Buffington and Liljeblad (2008), regardless of other material presenting more variable phenotypes. Buffington and Liljeblad (2008) established the length of the median mesoscutal impression as the single diagnostic character for these 2 species; our morphological data suggests that the 2-character states identified by Buffington and Liljeblad are instead 2 ends of a continuum (Figs 4 and 5). The relationship between the length of the median mesoscutal impression and body size, both of which are non-parametric, is very weakly monotonic as supported by a Spearman’s rank correlation test (ρ = 0.257, *P *= 0.022). Rather, the 2 variables have a weak positive correlation and are increasing concurrently but at different rates. Additional characters separating these 2 species were not identified by Buffington and Liljeblad, and our examination of other various characters on *Euceroptres* specimens (eg antennomere lengths and aspects of body sculpture) did not reveal noteworthy differences. As a result, we suggest that *E. primus* constitutes a single species exhibiting a moderate range of intraspecific phenotype variation. Based on the combination of molecular and morphological data suggesting the presence of only a single species within *E. primus* and *E. whartoni*, we establish *E. whartoni*  Buffington and Liljeblad, 2008 as a **syn. nov.** of *E. primus*  Ashmead, 1896.

## Discussion

Our results suggest 2 conclusions: (i) that *E. primus* are parasitoids, not inquilines, and (ii) that the 2 described species in eastern North America are a single species. Though *Euceroptres* were once assumed to be inquilines, we definitively show that they are primary parasitoids of oak gall wasp larvae. Micro-CT imaging of galls from which *Euceroptres* emerged shows only typical, unmodified chambers within those galls. Chambers in these galls did not dramatically differ from those inhabited by gall inducer in shape, size, or relative position. While this alone is not evidence for a parasitoid feeding ecology, the lack of atypical chambers within the pre-existing gall tissue, secondary modifications to the gall, or cavities from endophytophagy indicates that there was no inquiline presence. Therefore, *Euceroptres* do not adhere to the current definition of “inquilines” (Davis et al. 2026) and do not appear to cause any changes to the typical gall structure. Further, we discovered an *Euceroptres* adult in a typical gall wasp chamber and, most convincingly, a *Euceroptres* larva actively feeding on a larval gall inducer. The totality of evidence presented here clearly indicates that *E. primus* are primary parasitoids of the gall inducer. While it is unlikely that either *E. montanus* or *E. maritimus* has a different feeding ecology than *E. primus*, we did not directly study either of these western North American *Euceroptres* species.

The previous classification of *Euceroptres* as inquilines originates from phylogenetic reconstructions that used morphological features and inferences from known feeding ecologies of putatively sister taxa. Ronquist (1994) first suggested *Euceroptres* as “figitoid inquilines,” which were nested within Cynipidae. Because *Euceroptres* were included in this group, they were assumed to be phytophagous. Ronquist and Nieves-Aldrey (2001) transferred *Euceroptres* from Cynipidae to Figitidae but did not discuss the feeding ecology of the genus despite addressing the feeding ecology of a similar basal lineage, Parnipinae, which are koinobiont parasitoids of cynipid galls on Papaveraceae. More recently, Blaimer et al. (2020) published UCE phylogeny data that confirmed the placement of *Euceroptres* within Figitidae sensu lato and examined feeding ecology through ancestral state reconstructions. *Euceroptres* was coded as both parasitoids and unknowns in different versions of the analysis. However, all internal nodes of the broader clade containing *Euceroptres*, Liopteridae, Ibaliidae, and their relatives were recovered as parasitoids. Our finding of *Euceroptres* as a parasitoid is therefore parsimonious with expectations from these ancestral state reconstructions. This work highlights the importance of direct ecological observation in validating inferred traits. Future studies should aim to evaluate the feeding ecology of other gall-associated species, which have not yet been directly confirmed, such as *Ceroptres* Hartig, 1840; our methods establish a framework that may be broadly applied toward this objective.

Host ranges in the genus were not expanded much beyond what was previously known. The eastern species (now just called *E. primus*) has 9 hosts, while the western species still has just 1 known host each. It is possible that these species each have a larger host range, but considerable recent rearing of North American oak galls (eg Ward et al. 2022) suggests that *Euceroptres* species have more limited host ranges.

We also found that, contrary to our expectations, the genus *Euceroptres* was less species-rich than existing records implied. We found no evidence for cryptic or undescribed species, and, in fact, synonymized *E. whartoni* with *E. primus*, reducing the number of *Euceroptres* species from 4 to 3. The 2 western species, *E. montanus* and *E. maritimus*, were not studied in detail as part of this work; however, it is relevant to note that we observed moderate genetic differences at the mtCOI locus. Morphological characters separating *E. montanus* and *E. maritimus* are more robust than the single diagnostic character, which originally separated *E. primus* and *E. whartoni.* These traits include differences in the number of flagellomeres, the presence of felt-like setae at the mesopleural triangle, and sculpture differences at the metepisternum (Buffington and Liljeblad 2008).

The species-depauperate nature of *Euceroptres* raises questions about why some common oak gall-associated parasite genera are very species-rich and attack a large proportion of galls (eg *Ormyrus*, *Synergus*) while others are much less so and consequently attack fewer galls (eg *Euderus*, *Euceroptres*; Ward et al. 2019). Possible explanations range from differences in ecology among genera to differences in adaptive potential to historical perspectives (eg *Euceroptres* may be a “living fossil”; Buffington and Liljeblad 2008). Answering these questions may provide critical insight into the origins and maintenance of parasitic wasp diversity and thus are a fruitful avenue for future study. For *E. primus*, the host galls are predominantly sexual-generation, succulent, integral swellings of the leaf (Table 2). One possibility is that *E. primus* is specialized on a combination of specific gall phenotypic traits rather than being specialized on individual gall species. If so, divergent selection among populations using different host species may be limited.

Separately, the crypt-keeper wasp (Chalcidoidea: Eulophidae, *Euderus set*  Egan et al., 2017) has also been noted as an unusual gall parasitoid due to its use of a similar broad array of gall hosts, and it attacks many of the same species of fleshy, simple leaf galls from which *E. primus* was reared (Egan et al. 2017, Ward et al. 2019). The association of 2 polyphagous gall parasitoids from different superfamilies with this same array of hosts may inform future work on the relationship between galls and their parasites.

## Nomenclature

This paper and the nomenclatural act it contains have been registered in Zoobank (www.zoobank.org), the official register of the International Commission on Zoological Nomenclature. The LSID (Life Science Identifier) number of the publication is: urn:lsid:zoobank.org:pub:1986280E-684E-4160-BB1A-462AC10C45B1.

## Supplementary Material

saag028_Supplementary_Data

## Data Availability

MtCOI sequences generated through this work are available on GenBank (accessions PZ242853-PZ242882). New UCE sequence data are available in the NCBI Sequence Read Archive under Bioproject accession PRJNA1447577. Script for demultiplexing sequence data, creating distance matrices, performing statistics on morphometric data, and stylizing both the mtCOI and UCE trees are available on Zenodo (https://zenodo.org/records/19360684).
