# Supplementary material for: From two putative inquilines to one definitive parasitoid: clarifying feeding ecology and species limits in Nearctic Euceroptres Ashmead, 1896 wasps (Hymenoptera: Figitidae)
Source: Ann Entomol Soc Am. 2026 Jul 14;119(5):386–95. doi: 10.1093/aesa/saag028 (PMC13562751; doi:10.1093/aesa/saag028)
Supplement: Supplementary file 1 [file saag028_supplementary_data.zip › supplement_tables-S1-S3_&_figures-S1-S2.docx]

**Tables S1-S3**

**Figures S1-S2**

**for Brown et al. (****From two putative inquilines to one definitive parasitoid: clarifying feeding ecology and species limits in Nearctic *Euceroptres* Ashmead wasps (Hymenoptera: Figitidae)**)

Table S1. Settings for the climate-controlled incubator used to rear insects from oak galls, grouped by season.

| Season | Time | Temperature ºC | Relative Humidity | Lights |
| --- | --- | --- | --- | --- |
| Spring | 3:00am | 10 | 85 | off |
|  | 6:00am | 14 | 85 | on |
|  | 9:00am | 18 | 65 | on |
|  | 12:00pm | 22 | 75 | on |
|  | 3:00pm | 22 | 75 | on |
|  | 6:00pm | 18 | 80 | on |
|  | 9:00pm | 14 | 80 | off |
|  | 12:00am | 10 | 80 | on |
| Summer | 3:00am | 17 | 75 | off |
|  | 6:00am | 20 | 70 | on |
|  | 9:00am | 22 | 65 | on |
|  | 12:00pm | 24 | 70 | on |
|  | 3:00pm | 26 | 70 | on |
|  | 6:00pm | 24 | 70 | on |
|  | 9:00pm | 22 | 75 | off |
|  | 12:00am | 22 | 75 | off |
| Autumn | 3:00am | 5 | 75 | off |
|  | 6:00am | 10 | 70 | on |
|  | 9:00am | 14 | 70 | on |
|  | 12:00pm | 18 | 65 | on |
|  | 3:00pm | 10 | 65 | on |
|  | 6:00pm | 5 | 70 | on |
|  | 9:00pm | 5 | 75 | off |
|  | 12:00am | 5 | 75 | off |
| Winter | 3:00am | 5 | 75 | off |
|  | 6:00am | 5 | 70 | off |
|  | 9:00am | 10 | 70 | on |
|  | 12:00pm | 10 | 65 | on |
|  | 3:00pm | 10 | 65 | on |
|  | 6:00pm | 10 | 70 | off |
|  | 9:00pm | 5 | 75 | off |
|  | 12:00am | 5 | 75 | off |

Table S2. Morphometric data of 79 female *Euceroptres* specimens. The body length, mesoscutum length, and length of the mesoscutal impression are recorded in millimeters. The ratio of the impression to the mesoscutum is documented in the column named “imp_scut”.

| labCode | bodyLength_mm | mesoscutumL_LFN | mesoscutumL_mm | medianImpressionL_mm | imp_scut | generalColoration | host | sex |
| --- | --- | --- | --- | --- | --- | --- | --- | --- |
| 265-007-018 | 1.85 | 25 | NA | NA | 0 | black | Andricus foliaformis | female |
| 265-007-17 | 2.1 | 29 | NA | NA | 0.21 | black | Andricus foliaformis | female |
| 918-042-10B | 2.5 | 30 | NA | NA | 0.27 | black | Andricus quercuspetiolicola | female |
| 1426-010-17A | 2.2 | 28 | NA | NA | 0.29 | black | Andricus quercuspetiolicola | female |
| 1426-010-11B | 2.55 | 33 | NA | NA | 0.3 | black | Andricus quercuspetiolicola | female |
| 1426-010-11A | 2.2 | 29 | NA | NA | 0.28 | black | Andricus quercuspetiolicola | female |
| 1426-010-25A | 2 | 28 | NA | NA | 0.29 | black | Andricus quercuspetiolicola | female |
| 861-042-42B | 2.1 | 28 | NA | NA | 0.25 | dark brown | Andricus quercuspetiolicola | female |
| 762-042-17 | 1.65 | 22 | NA | NA | 0 | black | Andricus quercuspetiolicola | female |
| 918-042-13B | 1.9 | 27 | NA | NA | 0.19 | black | Andricus quercuspetiolicola | female |
| 1766-010-17D | 2.25 | 30 | NA | NA | 0 | black | Andricus quercuspetiolicola | female |
| 1776-010-15A | 1.7 | 27 | NA | NA | 0.3 | black | Andricus quercuspetiolicola | female |
| 1766-010-17C | 1.95 | 28 | NA | NA | 0.18 | black | Andricus quercuspetiolicola | female |
| 1763-010-4E | 2.6 | 29 | NA | NA | 0.17 | black | Andricus quercuspetiolicola | female |
| 1763-010-4G | 1.4 | 21 | NA | NA | 0.29 | black | Andricus quercuspetiolicola | female |
| 1782-010-4C | 1.15 | 13 | NA | NA | 0 | light brown | Andricus quercuspetiolicola | female |
| 1773-010-12B | 1.65 | 23 | NA | NA | 0.13 | black | Andricus quercuspetiolicola | female |
| 1763-010-6C | 2.4 | 31 | NA | NA | 0 | black | Andricus quercuspetiolicola | female |
| 1763-010-4C | 2.45 | 31 | NA | NA | 0 | black | Andricus quercuspetiolicola | female |
| 1776-010-13B | 2.35 | 29 | NA | NA | 0.24 | black | Andricus quercuspetiolicola | female |
| 1763-010-6D | 2.4 | 30 | NA | NA | 0.13 | black | Andricus quercuspetiolicola | female |
| 1769-010-19D | 2.2 | 30 | NA | NA | 0.2 | black | Andricus quercuspetiolicola | female |
| 1773-010-12A | 1.7 | 25 | NA | NA | 0.2 | black | Andricus quercuspetiolicola | female |
| 1766-010-10 | 2.3 | 32 | NA | NA | 0 | black | Andricus quercuspetiolicola | female |
| 1773-010-14A | 1.85 | 25 | NA | NA | 0.28 | black | Andricus quercuspetiolicola | female |
| 1771-010-5G | 2.2 | 27 | NA | NA | 0.07 | black | Andricus quercuspetiolicola | female |
| 266855 | 2.62 | NA | 0.54 | 0.07 | 0.13 | black | Andricus quercuspetiolicola | female |
| 266856 | 2.4 | NA | 0.5 | 0.07 | 0.14 | black | Andricus quercuspetiolicola | female |
| 267308 | 2.48 | NA | 0.52 | 0.05 | 0.1 | black | Andricus quercuspetiolicola | female |
| 267340 | 2.58 | NA | 0.5 | not perceptible | 0 | black | Andricus quercuspetiolicola | female |
| 267353 | 2.54 | NA | 0.52 | 0.07 | 0.13 | black | Andricus quercuspetiolicola | female |
| 267353 | 2.12 | NA | 0.4 | 0.1 | 0.25 | black | Andricus quercuspetiolicola | female |
| 267353 | 2.46 | NA | 0.4 | 0.07 | 0.18 | black | Andricus quercuspetiolicola | female |
| 862-013-009 | 1.2 | 15 | NA | NA | 0 | dark brown | Bassettia flavipes | female |
| 872-049-08A | 1.8 | 22 | NA | NA | 0.18 | black | Callirhytis scitula | female |
| 872-049-9A | 1.65 | 22 | NA | NA | 0.18 | black | Callirhytis scitula | female |
| 271883 | 2.63 | NA | 0.48 | 0.07 | 0.15 | black | Melikaiella tumifica | female |
| 271884 | 2.48 | NA | 0.5 | 0.11 | 0.22 | black | Melikaiella tumifica | female |
| 271885 | 2.08 | NA | 0.4 | 0.05 | 0.13 | black | Melikaiella tumifica | female |
| 271886 | 1.96 | NA | 0.48 | not perceptible | 0 | black | Melikaiella tumifica | female |
| 271887 | 2.47 | NA | 0.52 | 0.11 | 0.21 | black | Melikaiella tumifica | female |
| 271889 | 2.72 | NA | 0.56 | 0 | 0 | black | Melikaiella tumifica | female |
| 271890 | 2.52 | NA | 0.4 | 0.05 | 0.13 | black | Melikaiella tumifica | female |
| 271892 | 2.56 | NA | 0.54 | 0.07 | 0.13 | black | Melikaiella tumifica | female |
| 271893 | 2.4 | NA | 0.5 | 0.11 | 0.22 | black | Melikaiella tumifica | female |
| 271894 | 2.6 | NA | 0.38 | 0 | 0 | black | Melikaiella tumifica | female |
| 271895 | 1.76 | NA | 0.35 | 0 | 0 | black | Melikaiella tumifica | female |
| 271896 | 2.36 | NA | 0.45 | 0.07 | 0.16 | black | Melikaiella tumifica | female |
| 271897 | 2.48 | NA | 0.52 | 0.05 | 0.1 | black | Melikaiella tumifica | female |
| 271898 | 2.16 | NA | 0.37 | 0.1 | 0.27 | black | Melikaiella tumifica | female |
| 271899 | 2.46 | NA | 0.5 | 0.07 | 0.14 | black | Melikaiella tumifica | female |
| 271900 | 2.3 | NA | 0.46 | 0 | 0 | black | Melikaiella tumifica | female |
| 271901 | 2.16 | NA | 0.43 | 0.05 | 0.12 | black | Melikaiella tumifica | female |
| 271903 | 1.74 | NA | 0.36 | 0 | 0 | black | Melikaiella tumifica | female |
| 271904 | 2.24 | NA | 0.46 | 0.04 | 0.09 | black | Melikaiella tumifica | female |
| 271905 | 1.92 | NA | 0.4 | 0.07 | 0.18 | black | Melikaiella tumifica | female |
| 876-016-023 | 1.15 | 12 | NA | NA | 0 | light brown | Melikaiella ostensackeni | female |
| 876-016-24B | 1.45 | 18 | NA | NA | 0 | light brown | Melikaiella ostensackeni | female |
| 870-016-24E | 1.5 | 16 | NA | NA | 0 | light brown | Melikaiella ostensackeni | female |
| 810-043-7A | 2.25 | 26 | NA | NA | 0.31 | dark brown | Melikaiella tumifica | female |
| 810-043-7C | 2.25 | 29 | NA | NA | 0.21 | black | Melikaiella tumifica | female |
| 1383-097-18 | 1.2 | 14 | NA | NA | 0 | light brown | Melikaiella tumifica | female |
| 1383-097-15B | 1.25 | 14 | NA | NA | 0 | light brown | Melikaiella tumifica | female |
| 1383-097-15A | 1 | 12 | NA | NA | 0 | light brown | Melikaiella tumifica | female |
| 1383-097-14B | 1.25 | 14 | NA | NA | 0 | light brown | Melikaiella tumifica | female |
| whartoniHolotypeMale[USNMENT00802423] | 2.7 | 2.52 | NA | NA | 0.16 | NA | NA | female |
| primusHolotypeFemale[USNMENT00802259] | 1.8 | 3.97 | NA | NA | 0.17 | NA | NA | female |
| 269997 | 1.84 | NA | 0.4 | not perceptible | 0 | black | unknown | female |
| 269996 | 2.06 | NA | 0.38 | not perceptible | 0 | black | unknown | female |
| 269995 | 1.99 | NA | 0.4 | not perceptible | 0 | black | unknown | female |
| 269993 | 1.76 | NA | 0.38 | not perceptible | 0 | black | unknown | female |
| 269991 | 1.92 | NA | 0.38 | 0.07 | 0.18 | black | unknown | female |
| 269990 | 1.87 | NA | 0.35 | not perceptible | 0 | black | unknown | female |
| 269997 | 1.84 | NA | 0.4 | not perceptible | 0 | black | unknown | female |
| 269996 | 2.06 | NA | 0.38 | not perceptible | 0 | black | unknown | female |
| 269995 | 1.99 | NA | 0.4 | not perceptible | 0 | black | unknown | female |
| 269993 | 1.76 | NA | 0.38 | not perceptible | 0 | black | unknown | female |
| 269991 | 1.92 | NA | 0.38 | 0.07 | 0.18 | black | unknown | Female |
| 269990 | 1.87 | NA | 0.35 | not perceptible | 0 | black | unknown | Female |

Table S3. *Euceroptres* samples used for mtCOI phylogeny. The “Final Species Assignment” are the species names after synonymizing *E. primus* and *E. whartoni*.

| Sample | Final Species Assignment | Gall Host Species | Tree host | Collection (country:state) | Collection Location | Accession # | Representative Vouchered Sample(s) |
| --- | --- | --- | --- | --- | --- | --- | --- |
| 880-042-18A | Euceroptres primus | Andricus quercuspetiolicola | Quercus macrocarpa | USA:IA | Oxford_IA | PZ242853 | NA |
| 270-042-140 | Euceroptres primus | Andricus quercuspetiolicola | Quercus bicolor | USA:IA | Iowa-City_IA | PZ242854 | NA |
| 840-016-17B | Euceroptres primus | Melikaiella ostensackeni | Quercus velutina | USA:MO | Hannibal_MO | PZ242855 | NA |
| 840-016-23a | Euceroptres primus | Melikaiella ostensackeni | Quercus velutina | USA:MO | Hannibal_MO | PZ242856 | NA |
| 265-007-20A | Euceroptres primus | Andricus foliaformis | Quercus macrocarpa | USA:IA | Iowa-City_IA | PZ242857 | NA |
| 860-007-14E | Euceroptres primus | Andricus foliaformis | Quercus macrocarpa | USA:IA | Iowa-City_IA | PZ242881 | NA |
| 296-010-4 | Euceroptres primus | Andricus quercuspetiolicola | Quercus alba | USA:IA | Iowa-City_IA | PZ242879 | NA |
| 270-042-134 | Euceroptres primus | Andricus quercuspetiolicola | Quercus bicolor | USA:IA | Iowa-City_IA | PZ242858 | NA |
| 270-042-135 | Euceroptres primus | Andricus quercuspetiolicola | Quercus bicolor | USA:IA | Iowa-City_IA | PZ242859 | SUI_INS_49826 |
| 862-013-12A | Euceroptres primus | Bassetia flavipes | Quercus palustris | USA:IA | Iowa-City_IA | PZ242860 | NA |
| 862-013-12B | Euceroptres primus | Bassetia flavipes | Quercus palustris | USA:IA | Iowa-City_IA | PZ242882 | NA |
| 1371-051-5 | Euceroptres primus | Callirhytis quercusfutilis | Quercus bicolor | USA:IA | Iowa-City_IA | PZ242862 | SUI_INS_49827 |
| 1371-051-7 | Euceroptres primus | Callirhytis quercusfutilis | Quercus palustris | USA:IA | Iowa-City_IA | PZ242861 | NA |
| 872-049-7A | Euceroptres primus | Callirhytis scitula | Quercus rubra | USA:IA | Oxford_IA | PZ242863 | NA |
| 872-049-8B | Euceroptres primus | Callirhytis scitula | Quercus rubra | USA:IA | Oxford_IA | PZ242864 | NA |
| 816-016-24A | Euceroptres primus | Melikaiella ostensackeni | Quercus rubra | USA:IA | Oxford_IA | PZ242865 | NA |
| 870-016-24C | Euceroptres primus | Melikaiella ostensackeni | Quercus rubra | USA:IA | Oxford_IA | PZ242866 | NA |
| 840-016-23F | Euceroptres primus | Melikaiella ostensackeni | Quercus velutina | USA:MO | Hannibal_MO | PZ242867 | NA |
| 1383-97-13A | Euceroptres primus | Melikaiella tumifica | Quercus palustris | USA:IA | Iowa-City_IA | PZ242868 | NA |
| 810-043-7C | Euceroptres primus | Melikaiella tumifica | Quercus velutina | USA:IA | Oxford_IA | PZ242880 | NA |
| 1763-010-4-D1 | Euceroptres primus | Andricus quercuspetiolicola | Quercus bicolor | USA:IA | Iowa-City_IA | PZ242869 | NA |
| 1371-051-6B | Euceroptres primus | Callirhytis quercusfutilis | Quercus bicolor | USA:IA | Iowa-City_IA | PZ242877 | NA |
| 2347-10-G3-3 | Euceroptres primus | Andricus quercuspetiolicola | Quercus bicolor | USA:VA | Arlington_VA | PZ242870 | NA |
| 269-042-82 | Euceroptres primus | Andricus quercuspetiolicola | Quercus bicolor | USA:IA | Iowa-City_IA | PZ242871 | NA |
| 862-013-11A | Euceroptres primus | Callirhytis flavipes | Quercus bicolor | USA:IA | Iowa-City_IA | PZ242872 | NA |
| 810-043-7A | Euceroptres primus | Melikaiella tumifica | Quercus velutina | USA:IA | Oxford_IA | PZ242873 | NA |
| 2177-335-E4 | Euceroptres maritimus | Callirhytis quercussuttoni | Quercus agrifolia | USA: CA | San-Luis_CA | PZ242874 | SUI_INS_49825 |
| 880-042-25B | Euceroptres primus | Andricus quercuspetiolicola | Quercus velutina | USA:IA | Oxford_IA | PZ242875 | NA |
| 810-043-7B | Euceroptres primus | Melikaiella tumifica | Quercus velutina | USA:IA | Oxford_IA | PZ242876 | NA |
| 265-007-19 | Euceroptres primus | Andricus foliaformis | Quercus macrocarpa | USA:IA | Iowa-City_IA | PZ242878 | NA |

Figure S1. Unrpruned maximum likelihood phylogeny of mtCOI for *Euceroptres* built in IQ-TREE. Accession numbers are shown for all sequences obtained from genbank.

Figure S2. Unrpruned maximum likelihood UCE phylogeny for *Euceroptres* built in IQ-TREE. Accession numbers are shown for all sequences obtained from genbank.
